# REV1 Loss Triggers a G2/M Cell-Cycle Arrest Through Dysregulation of Mitotic Regulators

**DOI:** 10.3390/genes17010044

**Published:** 2025-12-31

**Authors:** Brailey Buntin, Madison Guyette, Vihit Gupta, Kanayo Ikeh, Sombodhi Bhattacharya, Erica N. Lamkin, Allison Lafuze, Roxana del Rio-Guerra, Jiyong Hong, Pei Zhou, Nimrat Chatterjee

**Affiliations:** 1 Department of Microbiology and Molecular Genetics, University of Vermont, Burlington, VT 05405, USA; 2 Sylvester Comprehensive Cancer Center, Miller School of Medicine, University of Miami, Miami, FL 33136, USA; 3 Larner College of Medicine, University of Vermont, Burlington, VT 05405, USA; 4 Department of Chemistry, Duke University, Durham, NC 27708, USA; 5 Department of Biochemistry, Duke University, Durham, NC 27710, USA; 6 University of Vermont Cancer Center, University of Vermont, Burlington, VT 05405, USA

**Keywords:** translesion synthesis (TLS), G2/M arrest, JH-RE-06, REV1, Cyclin B1, tubulins

## Abstract

**Background:** Genomic integrity is crucial to the cellular life cycle, which involves a tightly regulated process where cells progress through specific phases to ensure that fully replicated, undamaged DNA is inherited by daughter cells. Any dysfunction in this process or unrepaired DNA damage leads to cell cycle arrest and programmed cell death. Cancer cells are known to exploit these mechanisms to continue dividing. Usually, DNA damage arrests replication, allowing the DNA Damage Response (DDR) pathway to activate, which repairs the DNA or bypasses the damage to support cell survival and preserve genome integrity. For DNA damage bypass or translesion synthesis (TLS), a group of low-fidelity polymerases perform error-prone DNA synthesis opposite damaged bases, where REV1 functions as the main scaffolding protein. Previously, we reported non-TLS functions of REV1, including its role in triggering DNA damage-dependent specific DNA metabolic processes. **Methods and Results:** In this study, we demonstrate that REV1 plays a significant role in cell cycle progression and that its loss causes arrest at the G2/M phase in flow cytometry analysis. This unexpected phenotype includes dysregulation of G2/M regulators, such as Cyclin B1 and tubulins, in REV1-deficient cells compared to controls, as quantified by Western blot. Additionally, phosphorylation of histone H3 at serine 28 was significantly reduced in these REV1-deficient cells. These G2/M arrest features were even more pronounced in REV1-deficient cells treated with the tubulin inhibitor colchicine. **Conclusions:** Overall, this study reveals a previously unrecognized link between REV1 TLS polymerase inhibition and the G2/M cell cycle arrest.

## 1. Introduction

Although the core components that regulate and control the cell cycle are well understood, the cellular mechanisms that intricately link the cell cycle to genome stability remain only partially understood [1]. Genome instability, which is connected to DNA damage repair mechanisms and mutagenesis, is the fundamental cause of cellular dysfunction and disease [2]. In all living species, the cell cycle is a carefully ordered sequence of events that includes growth, DNA replication, and cell division, ensuring the accurate distribution of undamaged DNA into daughter cells [3]. In eukaryotes, especially vertebrates, a group of evolutionarily conserved proteins called cyclins (D, A, E, and B) are produced sequentially during the four phases of the cell cycle—G1, S, G2, and M, respectively—to bind and activate cyclin-dependent kinases (CDKs) [4]. The cyclin-CDK complexes assemble through a tightly regulated process to initiate and sustain phase-specific expression of downstream genes, while also promoting the expression of genes that protect the genome during each phase [4,5,6,7]. During the G1 phase, for instance, the cell cycle is reset through regulation of the Cyclin D-CDK4/6 complex following a previous division, establishing a new, ready state that responds to growth factor stimulation until the cell commits to pass the restriction (R) point [8,9]. Then, the Cyclin E-CDK2 complex drives cells past the R-point and prepares for entry into S-phase, a process further regulated by the Cyclin A-CDK2 complex, which mainly facilitates replication [10]. Finally, the Cyclin B-CDK1 complex, which accumulates during G2 to initiate mitosis by phosphorylating downstream molecules [11,12], is rapidly degraded during anaphase, allowing the cell to exit mitosis [13].

Moreover, chromatin, the dynamic histone–DNA complex, continuously undergoes assembly and disassembly, including transcriptional and post-transcriptional regulation of histone protein production and controlled chromatin modifications in a cell cycle-dependent manner [14]. More specifically, two key events characterize the global chromatin restructuring necessary for completing the cell cycle. First, new core histones are produced in early S phase, transcribed, and incorporated into newly synthesized DNA to form nascent chromatin, where essential histone post-translational modifications are re-established [15]. Second, during G2, nascent chromatin matures, and histone production ceases. During mitosis, chromosomes condense, chromatin remodeling and transcriptional complexes detach from chromatin, and the nuclear architecture, including chromatin domains or associations with the nucleus’s interior versus the periphery, breaks down [14,15].

Any DNA damage or abnormality during the cell cycle triggers an immediate arrest to facilitate repair or initiate programmed cell death [2], specifically when cell cycle abnormalities are directly associated with several debilitating diseases, from cancer to neurodegenerative disorders, cardiovascular disorders, and immunological disorders, among others [16]. Following DNA damage, a highly coordinated sequence of conserved events maintains genome integrity and prevents disease onset [2]. For example, among the cell cycle phases, the S-phase makes the genome most vulnerable to damage, because the DNA is single-stranded and unprotected during replication [2]. However, damage-specific DNA repair pathways effectively fix the damage and rescue stalled replication. For instance, single-base pair damage or damage spanning a few nucleotides is recognized by excision repair pathways–base excision repair (BER), mismatch repair (MMR), and nucleotide excision repair (NER)—which remove the damaged nucleotides and synthesize new DNA across the damaged site [2,5]. The BER pathway, which removes single-base lesions caused by oxidative damage, deamination, and alkylation, is most active during the G1 phase, with some replication-associated activity during the S phase and limited activity during G2/M phase of the cell cycle [17,18,19]. NER, on the other hand, repairs bulky DNA lesions primarily during S-phase; however, NER components are known to be upregulated in G1 and M phases of the cell cycle [20]. Similarly, MMR corrects base-base mismatches, including insertion/deletion loops generated during DNA replication or recombination, and mainly functions in the S phase, where at least two of its components are also expressed in G1 [20,21,22].

Similarly, DNA strand breaks, whether single-stranded (SSB) or double-stranded (DSB), are actively detected by damage-sensor kinases, ataxia-telangiectasia- and Rad3-related (ATR) or ataxia-telangiectasia-mutated (ATM), which initiate downstream signaling cascades to repair SSBs or DSBs [23]. For DSB repair, homologous recombination (HR) and non-homologous end joining (NHEJ) are two major repair mechanisms that fix the broken strands and help maintain genome integrity [2,5,23]. NHEJ is active throughout the cell cycle, but it is the main pathway for repairing DSBs in the G1 phase, when a sister chromatid is not available as an HR template. While NHEJ also remains functional in S, G2, and M phases, it is the dominant repair pathway for DSBs when HR is less active, [24]. Therefore, the availability of sister chromatids, which varies with the cell cycle, restricts HR to the S and G2 phases [25].

Remarkably, cancer cells are adept at manipulating specific repair pathways by overexpressing or suppressing key DNA repair genes to fix their own damage and support unchecked growth [26]. Similarly, in cancer cells, increasing strategic mutability—such as bypassing damaged DNA rather than repairing it via the translesion synthesis (TLS) pathway—can confer a growth advantage. Sometimes, this also helps cancer cells resist therapy by avoiding therapy-induced damage and escaping cell death [27,28,29]. For DNA damage bypass or TLS, a group of polymerases—REV1, POLζ_4_, POLκ, POLη, and POLι—enables the bypass of bulky DNA damage to continue replication and prevent cell death. Due to their open structure and lack of an exonuclease domain, DNA synthesis past damaged sites is typically low-fidelity, resulting in error-prone insertions and mutations [27,29]. Evolutionarily, this process promotes phenotypic diversity, but in diseases such as cancer, mutation formation can trigger disease onset and contribute to therapeutic resistance.

Furthermore, in recent years, TLS polymerases have been found to participate in other cellular pathways. For example, REV7, also known as MAD2B or MAD2L2, the accessory subunit of POLζ_4,_ is part of the 53BP1-shieldin complex at sites of DNA double-strand breaks [30]. Additionally, REV7 has high sequence similarity to the MAD2 spindle assembly checkpoint protein, and its depletion results in premature mitotic entry [31]. Therefore, it can serve as a key factor in determining whether repair is mutagenic or error-free across various contexts [32]. Similarly, the main scaffolding TLS polymerase, REV1, which coordinates the bypass of DNA damage by interacting with and recruiting other TLS polymerases to damage sites [33,34], is also involved in other cellular functions. From its classic role as a deoxycytidyl transferase [35], to maintaining trinucleotide repeat stability [36], to its roles in HR and NER [37,38], to its ability to unravel G4 quadruplexes [39], to regulating cell death responses in cancer cells [40,41], and its association with virus replication [42,43], REV1 has an expanded significance of its role in mutagenesis and genome instability. Like REV7, REV1 in yeast was found to be associated with the cell cycle, where it was enriched in the G2/M phase to address leftover post-replicative gaps from the S-phase [44]. However, the exact role of REV1 in the cell cycle is unknown. This study shows that REV1 depletion causes an unexpected G2/M arrest with characteristic features of transcriptional repression of G2/M cell cycle regulators, suppression of tubulin proteins and mitosis-specific histones, without affecting DNA synthesis during S-phase. These results have significant implications in reporting a new candidate, REV1, whose inhibition causes G2/M arrest—the most critical checkpoint in the cell cycle that controls genome integrity and prevents disease.

## 2. Materials and Methods

### 2.1. Cell Culture

Mouse embryonic fibroblasts (MEF), wild type (WT), and REV1 knockout (KO) cells were kindly provided by Neil deWind’s lab [45], and cultured at 37 °C with 5% CO_2_ in DMEM containing 4.5 g/L D-glucose (Gibco, Thermo Fisher Scientific, NY, USA). The media were supplemented with 10% (vol/vol) fetal bovine serum (FBS; Gibco, Thermo Fisher Scientific, Waltham, MA, USA) and 1% PenStrep (Gibco, Thermo Fisher Scientific, Waltham, MA, USA). The mouse *REV1*KO cells have a deletion of exon 10, which impairs REV1 function in MEF cells [45]. These cells will be referred to as “mouse *REV1*KO” cells in this study.

Similarly, human fibrosarcoma HT1080 cells (American Type Culture Collection (ATCC), MA, Virginia, USA) were cultured under the same conditions in RPMI with 4.5 g/L D-glucose (Gibco, Thermo Fisher Scientific, NY, USA), supplemented with 10% (vol/vol) fetal bovine serum (FBS; Gibco, Thermo Fisher Scientific, Waltham, MA, USA) and 1% PenStrep (Gibco, Thermo Fisher Scientific, Waltham, MA, USA). For trypsinization and downstream experiments, 0.25% trypsin-EDTA (Gibco) was used.

### 2.2. Western Blotting

Cells were lysed using RIPA lysis buffer (G-Bioscience, St. Louis, MO, USA) containing a protease and phosphatase inhibitor cocktail (Roche, Mannheim, Germany). Lysates were quantified with the Micro BCA Protein Assay Kit (Pierce, Thermo Fisher Scientific, Rockford, IL, USA) and then boiled for five minutes in 2×Laemmli Sample Buffer. After samples were run on a gel and transferred to a polyvinylidene difluoride membrane, the membranes were blocked with 5% Blocking Grade Blocker (Bio-Rad, Hercules, CA, USA). Primary and secondary antibodies were diluted in 5% BSA dissolved in 1× TBS with 0.01% Tween-20 (Fisher Scientific, Thermo Fisher Scientific, Waltham, MA, USA). The membranes were incubated overnight at 4 °C with the following primary antibodies: ꞵ-Actin (Cell Signaling Technologies, 13E5, Danvers, MA, USA) at 1:1000, Cyclin B1 (Cell Signaling Technologies, 4138T, Danvers, MA, USA) at 1:250, Phospho-Histone H3 (Cell Signaling Technologies, 9713, Danvers, MA, USA) at 1:250, Beta-Tubulin (Cell Signaling Technologies, 2128, Danvers, MA, USA) at 1:500, H3 (Cell Signaling Technologies, 4499, Danvers, MA, USA) at 1:250, and REV1 (Invitrogen, 48793, Carlsbad, CA, USA) at 1:1000. The membranes were washed four times in 1× TBST before incubation with a secondary antibody (LiCOR-Bio 800CW Goat Anti-Rabbit, LI-COR Biosciences, Lincoln, NE, USA) at 1:20,000. Imaging was performed with the LiCOR Odyssey Imager (LI-COR Biosciences, Lincoln, NE, USA). Protein expression levels were quantified using Image Studio relative to ꞵ-Actin and then normalized to experimental controls.

### 2.3. BrdU Staining for Flow Cytometry

BrdU staining was carried out following the manufacturer’s instructions (Invitrogen kit, 8811-6600, Thermo Fisher Scientific, Waltham, MA, USA). Specifically, 10^6^ cells were labeled with 10 μM BrdU for 45 min at 37 °C, then washed with Flow Cytometry Staining Buffer (provided with the kit; Invitrogen kit, 8811-6600, Thermo Fisher Scientific, Waltham, MA, USA). Next, 1× BrdU Staining Buffer was added to the cells, which were incubated for 15 min at room temperature in the dark. The cells were rewashed with Flow Cytometry Staining Buffer, then DNase I was added, and the samples were incubated in the dark for 1 h. After another wash, the cells were incubated with an anti-BrdU antibody conjugated to a fluorochrome for 30 min at room temperature in the dark. Data were acquired on a flow cytometer at the Flow Cytometry & Cell Sorting Facility, UVM. Data is presented as the total number of BrdU-positive cells detected by the flow cytometer.

### 2.4. FACS Analysis of Histone H3S28

2 × 10^6^ cells were trypsinized and centrifuged at 4000 rpm for 5 min. Pellets were washed three times with 1× PBS containing 2% FBS and fixed with ice-cold 70% ethanol for 30 min at 4 °C. Cells were then washed three times with 1× PBS and treated with 100 μg/mL of ribonuclease. Subsequently, DNA was stained with 50 μg/mL of Propidium Iodide and analyzed at the Flow Cytometry & Cell Sorting Facility at UVM for cell cycle analysis. An antibody against phosphorylated histone H3 at ser28 (EMD Millipore, 07-145, Burlington, MA, USA) at a 1:500 concentration was used to determine the percentage of cells in mitosis [46]. Percent H3pS28 positive cells were calculated by dividing the Alexa Fluor 647 (AF647) positive cells from the gated population by the total number of gated cells in the population.

### 2.5. Chemical Inhibitors and Drugs

100 nM Colchicine (Sigma Aldrich, Milwaukee, WI, USA) was used to incubate MEF wild type (WT) and *REV1*KO cells for 2 h at 37 °C with 5% CO2 in DMEM (ATCC), supplemented with 10% (*v*/*v*) FBS (Gibco) and 1% penicillin-streptomycin (Gibco). Colchicine disrupts microtubule formation and arrests cells in the G2/M phase of the cell cycle [47]; it was formulated and diluted according to the manufacturer’s instructions.

To inhibit REV1 activity, we used JH-RE-06 at 10 µM for various time points at 37 °C under atmospheric conditions, as discussed in the results section. JH-RE-06 was formulated in DMSO before being diluted to an effective concentration in DNase/RNase-free water (Thermo Fisher Scientific, 10977015, Waltham, MA, USA). JH-RE-06 (Supplementary Figure S1) is a potent REV1 inhibitor [48] that binds to the C-terminal Domain (CTD) and induces protein dimerization. This dimerization prevents the REV7 accessory subunit of the POLζ_4_ complex and inhibits TLS, as shown by decreased mutagenesis and increased cytotoxicity in DNA-damage-treated cancer cells [48]. Drs. Zhou and Hong laboratories synthesized this drug.

### 2.6. Real-Time RT-PCR

To quantify REV1 knockdown, siRNA-treated cells were pelleted, and RNA was isolated using the Zymo Research RNA Miniprep kit (Zymo Research Corporation, Irvine, CA, USA). RNA concentration was measured with a Nanodrop (Thermo Fisher Scientific, Wilmington, DE, USA). Approximately 10 ng of isolated RNA was analyzed for each reaction using the iTaq Universal SYBR Green One-Step Kit (Bio-Rad). The qPCR protocol included 50 °C for 10 min, followed by 95 °C for 1 min, then 40 cycles at 95 °C for 15 s and 60 °C for 1 min. Relative mRNA levels were determined by comparing detectable products above a basal threshold, and ΔΔCT values were calculated by normalizing to GAPDH RNA and then to the appropriate control [49]. Table 1 lists the primers used for REV1 RT-PCR reactions, with gene names in species-specific nomenclature.

### 2.7. Statistics

GraphPad Prism 10 was used for data analysis and statistical testing. FlowJo v10.10 software was employed for flow cytometry analysis. In all graphs, data are presented as means ± S.E.M. (standard error of the mean). All experiments included at least three independent replicates. Student’s *t*-test, two-way ANOVA, or the Kruskal–Wallis test for multiple comparisons correction was used to determine statistically significant differences between groups. A *p*-value < 0.05 was considered statistically significant. Detailed statistical information is provided in the figure legends.

## 3. Results

### 3.1. REV1 Knockout Cells Exhibit G2/M Cell Cycle Arrest Without Impacting S Phase

Generally, DNA damage, growth factor deficiency, errors in DNA replication, cellular stress, viral infections, or toxins activate checkpoints that pause the cell cycle for repair [9]. In general, an arrest in the G1 phase stops abnormal replication of damaged DNA; conversely, an arrest in the G2 phase prevents the separation of defective chromosomes—processes that depend on the activation of cell cycle checkpoints [2,9,50]. For example, p53-dependent p21 activation and Rb dephosphorylation initiate the G1 and G2 checkpoints in response to DNA damage [51,52,53], while nucleotide depletion, along with DNA damage, causes an S-phase arrest aimed at halting cell cycle progression and DNA duplication [54,55]. Similarly, SSB and DSB damage activate the ATR/CHK1 and ATM/CHK2 pathways, which inhibit cell cycle progression by phosphorylating key proteins, such as Cdc25 [56]. Additionally, cells possess evolutionarily conserved mechanisms that delay progression through mitosis until specific events are completed. For instance, the accurate segregation of chromosomes via spindle checkpoints, triggered by damage to mitotic spindles or unattached chromosomes to microtubules, prevents chromosome dysfunction—as demonstrated in studies with microtubule stabilizers that induce G2/M arrest in treated cells [57,58].

**Figure 1:**
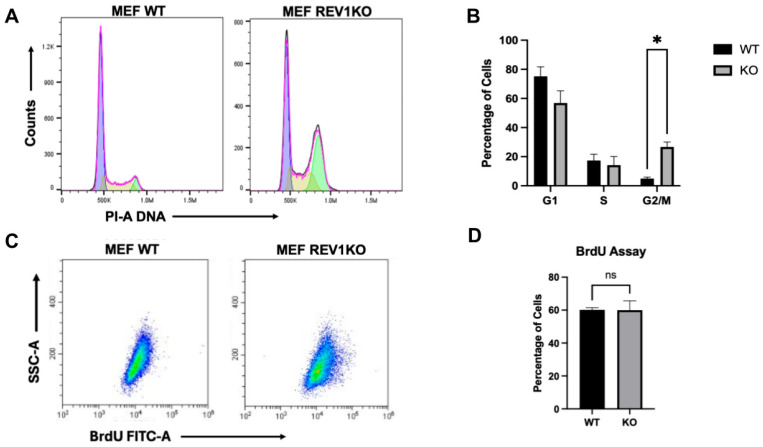
REV1 knockout cells are arrested in G2/M phase of the cell cycle. (**A**) Representative histograms represent cell cycle distribution in wild-type (WT) cells and in REV1 knockout (KO) Mouse Embryonic Fibroblasts (MEFs) following propidium iodide treatment in flow cytometry analysis. (**B**) Quantification of the different phases of the cell cycle from (**A**) in MEF WTs and *REV1*KO cells. Here, * *p*-Value = 0.0459, calculated by 2-way ANOVA, and error bars indicate mean ± standard error of mean (S.E.M.) (N = 3 biological replicates). (**C**) Representative dot-plots of the distribution of BrdU staining in cells of WT mouse embryonic fibroblasts (MEF) and *REV1*KO MEF cell lines in flow cytometry analysis. (**D**) Quantification of the percentage of cells from (**C**) stained with BrdU. Here, error bars represent the mean ± S.E.M. (N = 3 biological replicates), and the *p*-value calculated by a paired Student *t*-test, ns denotes non-significant. Statistical test done using Graphpad Prism 10.

The G2/M cell cycle arrest specifically prevents cells from entering mitosis when DNA damage or incomplete replication exists [59]. Here, ssDNA gaps formed spontaneously or remaining from the S-phase are filled by REV1-POLζ_4_, particularly in the G2 phase before cells enter mitosis [60,61]. This activity aligns with the observed increase in REV1 expression during the G2 phase in yeast models, where REV1 is reported to fill post-replicative gaps behind the fork, generated downstream of the lesion after repriming [44,62]. However, the effect of REV1 depletion on cell cycle progression remains unclear, despite prior studies using siREV1 showing some G2/M arrest only after ionizing radiation (IR)-induced DNA damage [63]. Figure 1A illustrates that mouse *REV1*KO cells, which lack REV1, show a significant G2/M arrest. Wild-type (WT) cells with functional REV1 cycle normally, with typical distribution across cell cycle phases. Conversely, mouse *REV1*KO cells exhibit a notable accumulation in G2/M-phase cells (Figure 1A,B), indicating that functional REV1 is necessary for cells to transition through G2/M and complete the cell cycle.

Furthermore, since REV1 is a DNA damage-bypass polymerase and is expected to bypass damage during DNA synthesis, we hypothesized that the absence of REV1 might hinder cells’ ability to bypass spontaneous damage, thereby promoting activation of the G2/M checkpoint arrest [64]. Therefore, mouse *REV1*KO cells may experience a slowdown in replication during S-phase. To test this, we stained cells with bromodeoxyuridine (BrdU) to accurately measure DNA synthesis using the BrdU assay (see Section 2 for details). Interestingly, we found that REV1-limited cells showed no difference in the overall amount of synthesized DNA (as indicated by BrdU incorporation) compared to wild-type cells with functional REV1 (Figure 1C), as demonstrated by flow cytometry-based dot blots and quantification of cells in the S-phase (Figure 1D). This suggests that, in REV1-limited cells, cell cycle checkpoints do not block or slow down replication but instead cause a G2/M arrest. We hypothesize that these REV1-limited cells might accumulate post-replicative gaps in G2/M due to the absence of REV1, leading to persistent DNA damage that triggers G2/M arrest, as reported by others [65].

### 3.2. REV1 Is Required for Cells to Progress Through the G2/M Phase of the Cell Cycle

Typically, during the G2 phase, cells continue their growth by producing essential proteins, engaging in organelle duplication such as mitochondrial duplication, preparing for mitosis by synthesizing microtubules that form the spindle, and, in cases of DNA damage or replication errors, triggering G2/M checkpoint arrest to maintain genome integrity [66]. The transition from G2 to M phase is enabled by the activation of the Cyclin B-CDK1 complex, also called the Maturation-Promoting Factor (MPF) [67], where Cyclin B transcription increases fourfold and translation rises up to twentyfold during G2 [68]. This promotes its binding to CDK1, which is phosphorylated at two key residues, Y15 and T14, by WEE1/MYT1, keeping MPF inactive [69]. However, at the end of G2, phosphorylated MPF, through a positive feedback loop, phosphorylates and inactivates WEE1/MYT1 and activates the CDC25C phosphatase, which dephosphorylates Y15 and T14, further activating CDK1 and committing cells to enter M phase [69]. At the end of M-phase, MPF activates the cyclin proteolytic degradation system, leading to its own inactivation. Additionally, another G2/M-specific regulator, Polo-like Kinase 1 (PLK1), ultimately regulates WEE1, MYT1, and CDC25C to promote mitotic entry [70]. Consequently, the expression of these key G2/M regulators is expected to be highly elevated during G2/M, gradually accumulating during the S phase, peaking in G2/M, and completing the cell cycle.

**Figure 2:**
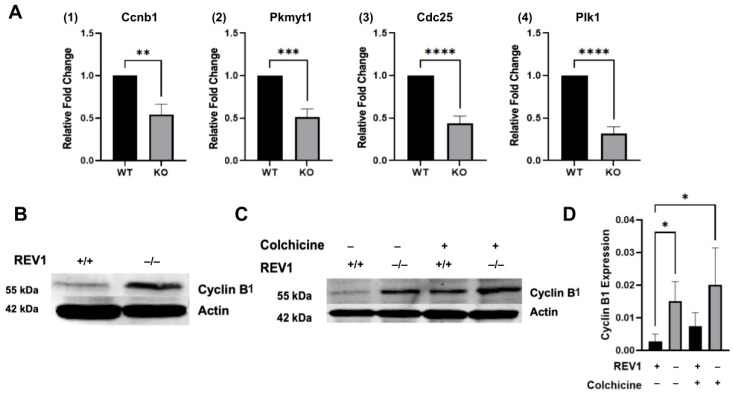
REV1 regulates expression of G2/M phase-specific biomarkers. (**A**) Relative quantification plots showing mRNA expression in the WT mouse embryonic fibroblasts (MEF) and *REV1*KO MEF cell lines (1) CcnB1, (2) Pkmyt1, (3) Cdc25, and (4) Plk1. Data presented as fold change relative to Wild-Type (WT) control. ** *p* < 0.0021, *** *p* < 0.0002, and **** *p* < 0.0001 calculated by unpaired *t*-test (N = 6 biological replicates). Gene names are in the mouse species nomenclature. (**B**) Representative Western blot of Cyclin B1 protein expression in WT mouse embryonic fibroblasts (MEF) and *REV1*KO MEF cell lines relative to β-actin. N = 3 biological replicates, quantification values in the Supplementary Materials Figure S2. (**C**) Western blots of Cyclin B protein expression in WT mouse embryonic fibroblasts (MEF) and *REV1*KO MEF cell lines relative to β-actin after exposure to 100 nM colchicine treatment for two hours. (**D**) Quantification plots showing relative expression of Cyclin B in WT mouse embryonic fibroblasts (MEF) and *REV1*KO MEF cell lines treated with Colchicine from (**C**). Data presented as mean ± S.E.M. (N = 3 biological replicates). * *p* < 0.05 determined via Kruskal–Wallis one-way ANOVA. Statistical test done using Graphpad Prism 10.

To characterize the REV1-loss-dependent G2/M arrest observed in Figure 1, we measured the transcript levels of key G2/M biomarkers—Cyclin B (CCNB1), PLK1, MYT1 (PKMYT1), and CDC25C—using qPCR in MEF cells. Figure 2A shows that in mouse *REV1*KO cells, the essential molecules needed for cells to transition from G2 to mitosis and complete cell division were transcriptionally downregulated. Not only was CcnB1, a key component of the MPF complex, repressed, but other critical G2/M phase regulators that activate the MPF complex, such as Plk1, Pkmyt1, and Cdc25C, also showed significant repression in mouse *REV1*KO cells (gene names in mice have the first letter capitalized, please see gene nomenclature description in the methods section). This transcriptional repression aligns with the idea that accumulation of DNA damage represses these genes, leading to G2/M arrest in some cancer cell models [71]. However, the decrease in Pkmyt1 is intriguing, as in typical G2/M arrest caused by DNA damage, Pkmyt1 levels usually increase to prevent entry into mitosis [72]. This suggests that REV1 may have an unexpected role in G2/M phase transition during the cell cycle.

To follow up on the transcript expression of G2/M regulators in REV1-limited cells, we measured the protein levels of the most significant G2/M molecule—Cyclin B1. Typically, Cyclin B1 levels can increase up to 20-fold during the cell cycle in normally cycling cells [73]. Figure 2B and Figure S2 show that Cyclin B1 levels in mouse *REV1*KO cells are elevated by up to 2-fold compared to wild-type cells. To further confirm the relatively small increase in Cyclin B1 protein in mouse *REV1*KO cells and to characterize its expression, we exposed mouse WT and *REV1*KO cells to low-dose colchicine, which induces G2/M arrest for about 2 h, and collected lysates without allowing recovery after treatment. Figure 2C,D demonstrate that Cyclin B1 levels are expectedly elevated in untreated mouse *REV1*KO cells, reaching the same level as colchicine-treated WT cells when compared to untreated WT cells. Additionally, colchicine-treated mouse *REV1*KO cells continue to show a significant increase in Cyclin B1 compared to untreated cells. However, this modest increase may be inconsequential, as its activity could be repressed by G2/M arrest and may be unable to form an active Cyclin B-CDK1 complex [74]. It is unknown how REV1 inhibition may be involved in the Cyclin B1 expression during the G2/M phase transition.

### 3.3. REV1 Knockout Cells Suppress Phosphorylation of Histone H3S28 and the Expression of Histone H3

**Figure 3:**
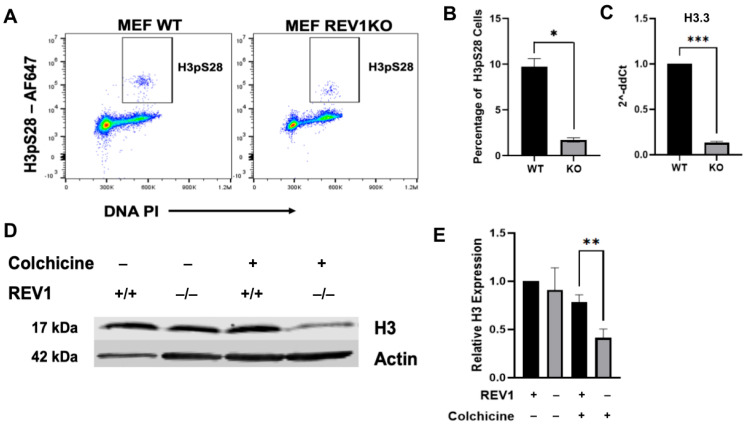
REV1 knockout cells exhibit reduced Histone 3 S28 phosphorylation and expression. (**A**) Cell cycle analysis with propidium iodide and Histone 3 pS28 staining to demarcate M-phase from the G2/M arrested in the WT mouse embryonic fibroblasts (MEF) and *REV1*KO MEF cells. Cells were analyzed on a flow cytometer after staining with propidium iodide and Histone 3 pS28. Percent Histone pS28 positive cells were calculated by dividing the Histone pS28 cells by the total cells. (**B**) Quantification of Histone 3 pS28 signal from (**A**). Data presented as mean ± S.E.M. (N = 3 biological replicates). * *p* < 0.01, calculated by paired Student *t*-test. (**C**) mRNA levels of Histone 3.3 tested by qPCR in mouse *REV1*KO cells. Data presented as fold change relative to Wild-Type (WT) MEF controls. *** *p* < 0.0002 calculated by Unpaired *t*-test, (N = 6 biological replicates). (**D**) Western blot analysis of WT mouse embryonic fibroblasts (MEF) and *REV1*KO MEF cell lines after exposure to 100 nM Colchicine for 2 h. (**E**) Quantification data from (**D**) is presented as mean ± S.E.M. (N = 3 biological replicates). ** *p* < 0.01 calculated by ordinary one-way ANOVA. Statistical test done using Graphpad Prism 10.

To explore the nature of the G2/M arrest observed in this study and determine whether REV1-limited cells were arrested in G2 or M phase, we examined Histone H3 S28 phosphorylation, a marker of the transcriptional response to cellular stress and mitosis [75,76]. H3S28 phosphorylation occurs specifically during early mitosis, coinciding with the start of mitotic chromosome condensation, mainly facilitated by Aurora B kinase, among other kinases [76,77]. During a normal transition from G2 into M phase, phosphorylation of H3S28 and H3S10 indicates appropriate cellular responses to stress [77], including entry into M phase, where chromosome condensation begins. Importantly, these phosphorylation events at H3S10 and H3S28 are not solely specific to the normal G2/M transition but are also involved in cellular responses to DNA damage such as UV radiation [78]. Therefore, we hypothesized that REV1-limited cells exhibiting G2/M arrest (Figure 1A,B), and possible spontaneous DNA damage, transcriptional repression of G2/M biomarkers (Figure 2A), and accumulation of non-degraded Cyclin B1 protein (Figure 2B–D) might have affected key histone expression and post-translational modifications critical for successful M-phase entry. We first assessed the relative expression of H3pS28 in mouse WT and *REV1*KO cells and found that, through FACS analysis, H3pS28 expression was significantly reduced in mouse *REV1*KO cells compared to WT (Figure 3A,B). Here, the percentage of cells expressing H3pS28 was calculated as the number of positive cells divided by the total number of cells. This result highlights a distinctive REV1-dependent G2/M arrest signature and suggests that REV1 deficiency impacts processes involved in chromosome condensation and cell cycle completion.

Intrigued by these M-phase-specific arrest mechanisms in Figure 3A,B, we wondered whether the general transcriptional expression of histone H3.3, a histone variant replicated independently of DNA replication and found in the chromatin of both active genes and silent regions [79], was also affected. Figure 3C shows that in mouse *REV1*KO cells, the mRNA levels of the histone variant H3.3 were significantly reduced compared to wild-type cells. This unexpected result suggests a broad dysregulation of both transcription and post-transcriptional modifications (PTMs) of histones and their variants in the REV1-inhibition-mediated G2/M arrest. To further confirm whether the overall expression of the H3 histone and its variants, H3.1 and H3.2, was similarly repressed, we tested protein levels in mouse *REV1*KO cells compared to WT cells. We found that replication-dependent (RD) histone variants were not repressed in mouse *REV1*KO cells relative to controls (Figure 3D). However, when cells were treated with colchicine, which induces G2/M arrest, H3 levels remained unchanged in wild-type cells, but in mouse *REV1*KO cells, H3 expression was significantly decreased (Figure 3D,E). This result indicates that *REV1*KO cells, already arrested at the G2/M phase, do not cycle sufficiently through the cell cycle to produce S-phase-specific, or replication-dependent, histone variants H3.1 and H3.2.

### 3.4. REV1 Regulates Tubulin Expression

**Figure 4:**
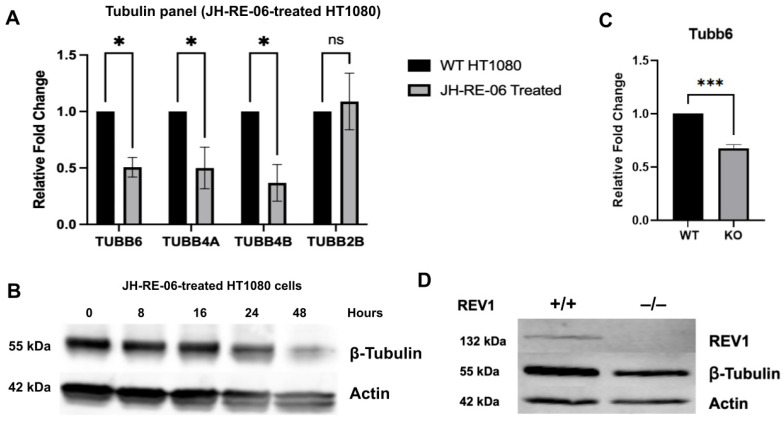
REV1 inhibition suppresses tubulin expression. (**A**) mRNA levels of β-Tubulin genes in HT1080 cells treated with REV1 inhibitor JH-RE-06 were measured using qPCR. Results are shown as mean fold change ± SEM (N = 3 biological replicates) compared to untreated cells. * *p* < 0.02, calculated using unpaired Student’s *t*-test, ns denotes non-significant. (**B**) Representative Western blot of β-tubulin expression relative to β-actin in HT1080 cells treated with JH-RE-06 for 8, 16, 24, and 48 h. Quantification of data is in the Supplementary Materials. (**C**) mRNA levels of Tubb6 in WT mouse embryonic fibroblasts (MEF) and *REV1*KO MEF cells using qPCR. Results are shown as mean fold change ± S.E.M. (N = 6 biological replicates) compared to untreated cells. *** *p* < 0.0002, calculated using unpaired Student’s *t*-test. Statistical test done using Graphpad Prism 10. (**D**) Representative Western blot of REV1 and β-Tubulin protein expression in WT mouse embryonic fibroblasts (MEF) and *REV1*KO MEF cell lines relative to β-actin. N = 3 biological replicates, quantification values in the Supplementary Materials. Gene names are in the mouse species nomenclature.

Intrigued by our observations of colchicine-dependent upregulation of Cyclin B1 and downregulation of H3 in mouse *REV1*KO cells (Figure 2C,D and Figure 3D,E), we questioned whether REV1 limitation might also affect tubulin expression dynamics. Tubulins are essential components of the eukaryotic cytoskeleton, where a family of globular proteins forms microtubule subunits that enable cargo transport by motor proteins and create mitotic spindles needed for chromosome separation during cell division [80]. Specifically, colchicine is a known microtubule inhibitor that binds to the soluble tubulin heterodimer, preventing its assembly into microtubules [81]. By blocking microtubule assembly, colchicine halts cell division during mitosis and induces G2/M arrest [81,82]. To explore a potential link between REV1 limitation and tubulin gene expression, we first measured mRNA levels of select human β-tubulin genes—TUBB6, TUBB4A, TUBB4B, and TUBB2B—in HT1080 cells. These tubulin genes are known targets of colchicine and other microtubular toxins, and their inactivation impairs the cell cycle by causing G2/M arrest [83], due to defective microtubules and failed chromosome segregation. Figure 4A displays a distinct pattern of tubulin expression in human HT1080 cells treated with REV1 inhibitor JH-RE-06 for 24 h. Here, TUBB6, TUBB4A, and TUBB4B are downregulated at the mRNA level in REV1-inhibited cells compared to controls, while TUBB2B levels remain unchanged. It is unclear how the REV1 limitation prompts changes in tubulin gene expression. To confirm our findings, we examined endogenous total β-tubulin protein levels via Western blot. In a time-course experiment, REV1 inhibition by JH-RE-06 over 48 h—starting at 8 h—gradually decreased β-tubulin protein levels. Figure 4B and Figure S3 show that β-tubulin expression is significantly reduced at 48 h, indicating that REV1 inhibition-dependent G2/M arrest exhibits β-tubulin dysregulation. Likewise, we probed β-tubulin expression in mouse *REV1*KO cells at both the transcript and protein levels to confirm our observations from inhibiting REV1 in human HT1080 cells. Figure 4C shows that Tubb6, one of the downregulated mRNAs in Figure 4A, was also repressed in the mouse *REV1*KO cells. Furthermore, when β-tubulin expression was probed by Western analysis in mouse *REV1*KO cells, which lack REV1 expression, β-tubulin expression was significantly downregulated, as shown in Figure 4D and Figure S4, suggesting that β-tubulin protein expression is dysregulated in REV1-limited cells.

## 4. Discussion

This study reports that REV1 TLS polymerase limitation causes cell cycle arrest in the G2/M phase, and that cells with a nonfunctional REV1 show a notable increase in G2/M phase compared to controls (Figure 1A,B). Interestingly, other components of the TLS pathway have been previously linked to conflicting roles in the cell cycle. For example, deleting yeast REV7, the accessory subunit of POLζ_4_, does not lead to cell cycle arrest [84]. However, in HeLa cells, REV7 was shown to be necessary for mitotic spindle organization and proper chromosome segregation, and depleting REV7 with siRNA increases the number of cells in both the S and G2/M phases [85]. Similarly, knockdown of REV7, REV3, and REV1 using siRNA in HeLa cells enhanced G2/M arrest induced by ionizing radiation, without affecting the cell cycle in individual experiments [63]. In addition, shREV3 knockdown in various cancer cell models amplified G2/M arrest, depending on the p53 status [86]. Moreover, REV7 has been shown to help maintain the bistability of the anaphase-promoting complex/cyclosome (APC/C)^CDC20^ and (APC/C)^CDH1^ complexes during the metaphase-to-anaphase transition, thus supporting mitotic accuracy [87]. This indicates a significant role for REV7, also known as Mitotic Arrest Deficient 2 Like 2 (MAD2L2), in regulating G2/M phase. The reason for the differing REV7 associations between yeast and human cell models remains unclear. Additionally, the role of REV1, the primary scaffolding molecule in the TLS pathway, was previously undefined. This study clarifies that limiting REV1 causes a pronounced G2/M arrest. The effect of REV1 deficiency in leading cells to arrest at G2/M has also been recently observed in REV1-overexpressing cells, which mimic REV1 limitation and cause G2/M arrest [88].

We also observe no difference in BrdU staining between mouse WT and *REV1*KO cells (Figure 1C,D), suggesting that REV1 deficiency does not affect DNA replication rates. This result suggests that REV1 may play a significant role in the G2/M transition, which has minimal impact on the S-phase, as seen by a lack of differences in the BrdU staining. Alternatively, REV1-limited cells might accumulate more DNA damage toward the end of S-phase, leading to activation of G2/M checkpoint signaling. Clearly, these potential mechanisms could contribute to the lower proliferation rates observed in *REV1*KO cells [40]. Additionally, as seen in yeast models, the increased expression of REV1 during the G2 phase of the cell cycle explains the absence of an S-phase-specific effect, as its function may be crucial for addressing post-replicative gaps in G2/M [44]. Increased gaps may accumulate DNA damage and cause G2/M arrest. Interestingly, the REV1 inhibitor JH-RE-06 was recently shown to directly increase γH2AX in a colorectal cancer cell model [65], even though REV1 inhibition through JH-RE-06 does not directly elevate γH2AX in vivo during chemotherapy-induced damage [40], indicating the involvement of other cellular response mechanisms in chemotherapy damage versus post-replicative gap filling of undamaged DNA by REV1.

If DNA damage is detected, the cell pauses to repair it before moving through any cell cycle phase. During the G2/M phase, this usually involves suppressing key regulators, triggering a G2/M checkpoint that stops cells from entering M phase. Figure 2A shows that transcriptional repression of key G2/M regulators—Cccnb1, Pkmyt1, Plk1, Cdc25C—in mouse *REV1*KO cells indicates that the absence of REV1 might cause accumulation of double-strand breaks due to unaddressed post-replicative gaps. Typically, DNA damage prevents the Cyclin B-CDK1 complex, essential for mitotic entry, from functioning by activating damage-response kinases such as ATM and ATR, which then phosphorylate and activate effector proteins such as CHK1 and CHK2. These effectors phosphorylate the phosphatase Cdc25C, sequestering it by 14-3-3 proteins and preventing it from activating the Cyclin B-Cdk1 complex [89]. Additionally, DNA damage heavily depends on p53 activation, which can repress CCNB1, PKMYT1, PLK1, and CDC25C, leading to a G2/M arrest [71]. Since REV1 is known to regulate p53 expression [90], it remains unclear whether REV1 influences G2/M regulators by modulating p53. Moreover, the unusual downregulation of PKMYT1 in REV1-limited cells suggests these cells may prematurely enter mitosis (M phase) without fully repairing their DNA [91].

Interestingly, the Cyclin B1 protein level, which peaks at up to 20-fold in normal G2/M-transiting cells [68], was about 2.5-fold in mouse *REV1*KO cells or after colchicine treatment (Figure 2B–D). While it is known that specific post-transcriptional regulatory mechanisms, such as those reported in the Drosophila model, repress translation by sequestering existing Cyclin mRNA [92], these mechanisms might explain the low mRNA and high protein levels of Cyclin. However, how a TLS polymerase participates in this remarkable biology remains unknown. Additionally, Cyclin B peaks during G2 phase, forming a complex with CDK1, which is rapidly degraded in mitosis to allow cell exit from mitosis. For successful exit and entry into the G1 phase, the Anaphase-Promoting Complex (APC/C) targets Cyclin B for quick degradation via ubiquitination [93]. As shown in Figure 2C,D, mouse *REV1*KO cells with an existing G2/M arrest continue to show Cyclin B1 protein expression despite the suppression of Ccnb1 mRNA in mouse cells in Figure 2A. Alternatively, since Cyclin B1 has two main transcripts—one constitutively expressed and the other mainly active during G2-M phase [94]—the roughly 2-fold increase in Cyclin B1 protein in mouse *REV1*KO cells could simply be an accumulation of the constitutively expressed transcript, which is translated but not yet degraded by the APC due to the G2/M arrest. This fascinating biology warrants further investigation.

Additionally, cells produce large amounts of replication-dependent (RD) histones during S-phase to package newly replicated DNA, ensuring proper chromatin organization and distribution to daughter cells [95]. Among core histone proteins—H2A, H2B, H3, H4, and the H1 linker—they form octamers that wrap DNA into nucleosomes. Histones also exist as variants, encoded by different genes with similar protein sequences [96]. The presence of multiple variants for a single histone type, such as RD-specific H3.1 and H3.2, and replication-independent variants like H3.3, adds an extra layer of chromatin regulation by diversifying nucleosome structure, function, and stability. This influences histone post-translational modifications (PTMs) and the recruitment of chromatin-associated proteins, significantly affecting transcription and cell fate decisions [96]. During the G2/M phase of the cell cycle, histone variants and PTMs ensure accurate chromosome segregation and signal spindle formation [97]. Our observation that Histone H3 S28 phosphorylation, a marker of mitosis [75,76], was reduced in mouse *REV1*KO cells (Figure 3A,B) highlights that the absence of REV1 arrests cells at the G2/M phase and indicates that REV1 may play a key role in the transition from G2 to M phase. However, further experiments that confirm differences in chromosome condensation may shed more light on the underlying biology.

Unexpectedly, in our effort to profile the overall expression of replication-dependent and -independent histone variants, we uncovered an interesting REV1-dependent histone regulatory biology. First, we found that mouse *REV1*KO cells have significantly lower mRNA levels of the H3.3 variant (Figure 3C). Typically, H3.3 mRNA expression remains stable throughout the cell cycle, with relatively low levels during the entire cycle [98]. This low-level H3.3 expression provides a ready histone replacement mechanism, since it is incorporated replication-independently into chromatin at active transcription sites, DNA repair regions, and other regulatory regions such as promoters, enhancers, telomeres, and centromeres, to sustain an active chromatin state [99]. Given that downregulation of the H3.3 gene in mice increases G2/M arrest, yet nocodazole-induced G2/M arrest does not elevate H3.3 levels, it is hypothesized that proper H3.3 levels are essential for normal G2/M progression [100]. Thus, the decreased H3.3 mRNA levels in mouse *REV1*KO cells suggest that REV1 may have a specific role in maintaining H3.3 variant expression, or that excess DNA damage due to limited REV1 function suppresses H3.3, both of which warrant further investigation. Second, our observation that colchicine treatment of mouse *REV1*KO cells suppresses H3 (H3.1 and H3.2) expression, unlike in wild-type cells, again indicates a unique REV1-dependent regulation of the cell cycle. Since colchicine is not known to influence H3 production [101], the mechanism behind H3 downregulation in mouse *REV1*KO cells remains unclear. Lastly, another unexpected REV1-dependent, G2/M-associated biological process we identified is that, in REV1-limited cells, β-tubulin gene expression at both the mRNA and protein levels was dysregulated, the mechanism of which is unknown (Figure 4A–D). In addition to direct mitotic inhibitors such as colchicine and taxol-based drugs, other factors, including tubulin monomer autoregulation, cellular differentiation, and external stress, can disrupt tubulin gene expression [102,103]. How the TLS polymerase REV1 regulates the expression of key G2/M biomarkers remains unknown and warrants further investigation. Whether REV1-limitation indirectly regulates checkpoints via replication stress signaling and chromatin assembly, instead of a direct regulatory role, also needs to be investigated. However, this study expands the significance of REV1’s role in cell cycle biology, specifically by triggering a G2/M arrest, with clinical implications for diseases, specifically cancer pathogenesis and resistance, given that REV1 targeting by the JH-RE-06 inhibitor is known to combat cancer chemoresistance [48], as reported for other tumor suppressor candidates [104,105].

## 5. Conclusions

**Figure 5:**
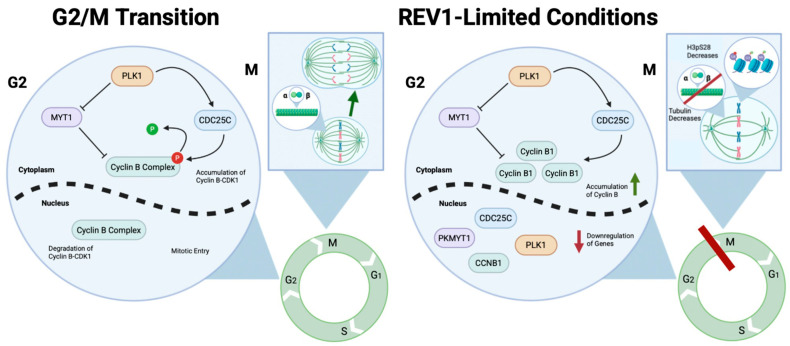
Model showing REV1’s role in regulating the G2/M transition and specifically the completion of M-phase. (**Left**) The sequence of events that enables wild-type cells to move from G2 to M phase is shown in the blue circle on the left (which serves as an inlet from the cell cycle depicted in green, showing the G1, S, G2, and M phases). During the G2 phase, upregulation of Cyclin B1 transcription and translation allows it to form a complex with CDK1, creating the Maturation Promoting Factor (MPF), which remains inactive due to phosphorylation (shown as a red P) by G2/M-specific kinases—MYT1 and WEE1—along with PLK1, and the phosphatase CDC25. Here, PLK1 inhibits MYT1, which in turn inhibits the MPF complex. PLK1, through CDC25C, also helps maintain the negative phosphorylation on the MPF complex. At the end of G2, MPF, through positive feedback, phosphorylates and inhibits the G2/M biomarkers to initiate mitosis, after which they are rapidly degraded during M phase. Also shown in the rectangle is an inlet from the M phase of the cell cycle, where tubulin proteins control the normal segregation of chromosomes. (**Right**) In the absence of REV1, a G2/M cell cycle arrest occurs (represented as a red rectangle blocking the G2/M phase in the green circle representing the cell cycle), characterized by repression of gene expression of the G2/M biomarkers (PKMYT1, CCNB1, PLK1, and CDC25 phosphatase), with minimal, but evident increased protein accumulation of Cyclin B1, decreased histone expression and histone variant PTMs, and dysregulated expression of tubulin genes. Downregulation of H3S28 phosphorylation, along with possibly disrupted chromosomal segregation, due to REV1 limitation, is shown in the rectangle, an inlet from the cell cycle represented as a green circle. Arrows indicate the next step in the pathway; inhibitory arrows indicate negative suppression of the downstream molecules; red arrows indicate suppression; green arrows indicate increased expression; PLK1 is in orange; PKMYT1 (gene) and MYT1 (the corresponding protein) is shown in purple; CDC25C is in blue; CCNB1 (gene) and Cyclin B1 (protein) is in green.

This study reveals an unexpected REV1-dependent regulation of the G2/M phase of the cell cycle (Figure 5). Unlike previous reports on REV7 or limited studies on TLS inhibition using siRNA, this is the first study to demonstrate that REV1 is essential for the proper transition from G2 to M phase. Additionally, we identify a unique G2/M arrest mechanism in the absence of REV1. Typically, as cells approach the end of G2, the Cyclin B–CDK1 complex, known as the mitotic promoting factor (MPF), accumulates in the cytoplasm and remains inactive due to inhibitory phosphorylation of specific CDK1 residues by Wee1 and Myt1, respectively [106]. Here, Polo-like kinase 1 (PLK1) phosphorylates CDC25, promoting its nuclear import [107,108]. Once the MPF complex accumulates in the nucleus, mitosis is initiated, and MPF is marked for degradation during late anaphase by ubiquitination of Cyclin B through the anaphase-promoting complex (APC) [109]. Similarly, tubulin-mediated spindle formation and subsequent chromosome separation during mitosis occur during the G2/M transition. However, REV1-limited cells (Figure 5) show delayed mitosis, as indicated by reduced Histone H3 phosphorylation at Serine 28, suggesting reduced mitotic entry. Cyclin B1 accumulates despite the delay in mitosis. There is also downregulation of genes coding for regulatory proteins such as MYT1, PLK1, and CDC25. Moreover, decreases in tubulin protein levels and gene expression are observed; thus, REV1 plays a previously unknown role in regulating the G2/M transition and cell entry into mitosis by involving several key components, whose mechanisms require further clarification.

Limitations of this study: This research provides the first evidence that inhibition of the primary scaffolding molecule of the TLS pathway, REV1, induces a G2/M cell cycle arrest characterized by altered expression of critical components of G2/M phase biomarkers. However, the detailed molecular mechanism remains unknown. For instance, it is unclear whether REV1 influences the expression of cyclin-dependent kinases (CDKs) active during other cell cycle phases. A comprehensive analysis of cyclins and CDKs at different stages of the cell cycle to confirm REV1’s broader regulatory role has not been conducted. Additionally, the relative quantification of REV1 expression across various cell cycle phases is unknown. Such data could help determine if REV1’s own expression is a key component of the suppressed network of G2/M biomarkers. Third, future research should assess protein expression of all G2/M biomarkers, including verification of other histone variants and histone PTM modifiers like H3pS10. Fourth, future studies should examine the relationship between REV1 and tubulin expression and stability, as the mechanism behind this is not addressed in this study. Finally, this study has some technical limitations, such as the use of mouse embryonic fibroblasts (MEFs) and HT1080 fibrosarcoma cells. Whether other cell types or disease models exhibit similar cell cycle defects upon REV1 removal is unknown. Further, this study does not examine the clonality of these cell lines, such as aged or young cells, to quantify the impact of REV1 deficiency on the cell cycle with clonal expansion. Further studies are needed to quantify REV1 deficiency-dependent G2/M arrest in long-term growth phenotypes or in vivo animal studies.

Furthermore, understanding how cancer cell evolution—predicted to follow a two-phase process of micro- and macroevolution through pathways of genome instability—contributes to the unique REV1-dependent cell cycle biology requires further investigation [110,111,112], specifically examining how (a) small-scale genomic changes in cancer microevolution, such as point mutations or insertions/deletions, provide an initial growth and clonal expansion advantage through DNA repair and cell cycle regulation, thereby promoting early cancer heterogeneity, and (b) how larger modifications during cancer macroevolution, like chromosomal rearrangements over time, are crucial in breaking system-level constraints that enable the emergence of new cellular systems—often manifested as the appearance of novel karyotypes—and thereby impact REV1-limited cells and their effects on the cell cycle. These different evolutionary stages, along with the cell-type and time-specificities of cancer cells, are important avenues for future research to fully understand how REV1 limitation over time fundamentally influences cancer versus normal cell-cycle dynamics.

## Figures and Tables

**Table 1:** Primer sequences *.

Gene	Forward Sequence	Reverse Sequence
GAPDH	CTGTTGCTGTAGCCAAATTCGT	ACCCACTCCTCCACCTTTGAC
Gapdh	CATCACTGCCACCCAGAAGACTG	ATGCCAGTGAGCTTCCCGTTCAG
CcnB1	AGAGGTGGAACTTGCTGAGCCT	GCACATCCAGATGTTTCCATCGG
H3.3	ACAAAAGCCGCTCGCAAGAGTG	TTCTCGCACCAGACGCTGAAAG
Pkmyt1	GGTCTCACCATCTTGGAAGTGG	CAGCATCATGGCGAGGACAGAA
Plk1	CCATCTTCTGGGTCAGCAAGTG	CCGTCATTGTAGAGAATCAGGCG
TUBB6	TGGACTTAGAGCCAGGCACCAT	TTTCGCCCAGTTGTTCCCTGCA
TUBB4A	CAGTGACGAACATGGCATCGAC	AGCACCGCTCTGGGGACATAAT
TUBB4B	TTGGGAGGTGATCAGCGATGAG	CTCCAGATCCACGAGCACGGC
TUBB2B	GCACGATGGATTCGGTTAGGTC	TCGGCTCCCTCTGTGTAGTGG
Tubb6	CGAGGCACAATGGACTCAGTCA	TGCCCAGTTATTTCCTGCACCAC

* Gene names in humans are capitalized, and in mice, the first letter is capitalized.

## Data Availability

The original contributions presented in this study are included in the article/Supplementary Material. Further inquiries can be directed to the corresponding author.

## Supplementary Materials

The following supporting information can be downloaded at: https://www.mdpi.com/article/10.3390/genes17010044/s1. Figure S1: Structure of JH-RE-06.NaOH, Figure S2: Relative expression of Cyclin B1 protein in the mouse embryonic fibroblasts *REV1*KO cells compared to the WT controls, Figure S3: Tubulin expression (JH-RE-06-treated HT1080), Figure S4: Relative expression of β-tubulin protein in the mouse embryonic fibroblasts *REV1*KO cells compared to the WT controls.
