# Supplementary material for: REV1 Loss Triggers a G2/M Cell-Cycle Arrest Through Dysregulation of Mitotic Regulators
Source: Genes (Basel). 2025 Dec 31;17(1):44. doi: 10.3390/genes17010044 (PMC12841074; doi:10.3390/genes17010044)
Supplement: Supplementary file 1 [file genes-17-00044-s001.zip › genes-4037589-supplementary.pdf]

Supplementary Figure 1

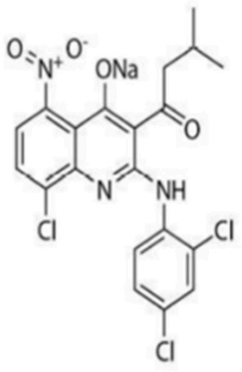

JH-RE-06.NaOH Salt form

Supplementary Figure 1: Structure of JH-RE-06.NaOH

Supplementary Figure 2

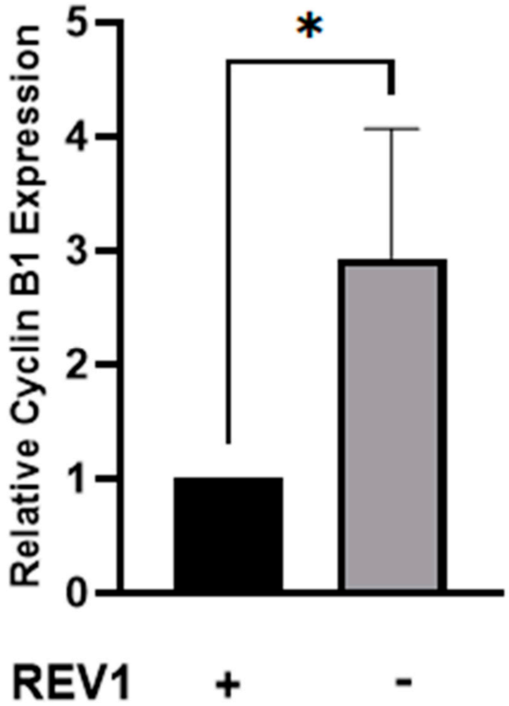

Supplementary Figure 2: Relative expression of Cyclin B1 protein in the mouse embryonic fibroblasts REV1KO cells compared to the WT controls. The graph represents quantified data from Fig. 2B. Results are shown as mean ± S.E.M. (N = 3 biological replicates). *P<0.01 values calculated by ***unpaired Student's t-test***. Statistical test done using Graphpad Prism 10.

Supplementary Figure 3

Tubulin expression (JH-RE-06-treated HT1080)

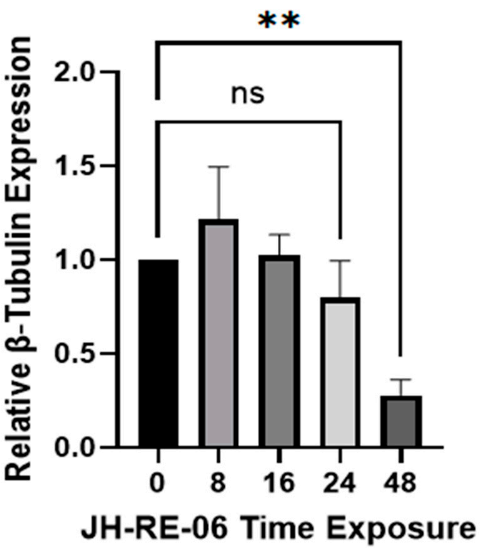

Supplementary Figure 3: Relative quantification of β-tubulin protein in HT1080 cells treated with JH-RE-06 for the indicated time course of 0, 8, 16, 24, and 48 hours, compared to controls from data presented in **Figure 4B**. Results are shown as mean ± S.E.M. (N = 3 biological replicates). **P<0.002 values calculated by ***ordinary One-way ANOVA***. Statistical test done using Graphpad Prism 10.

Supplementary Figure 4

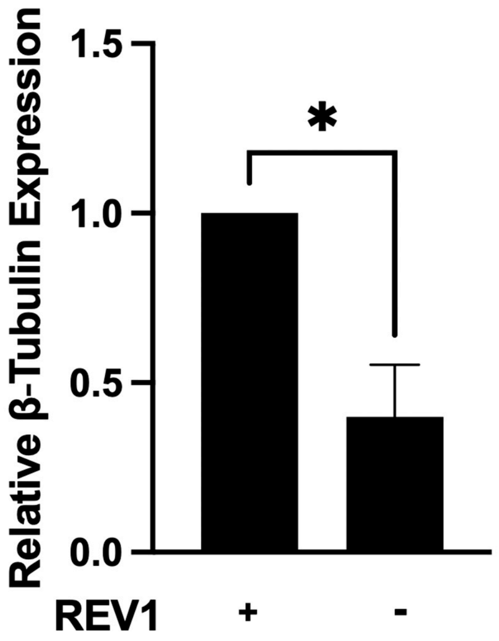

Supplementary Figure 4: Relative expression of β-tubulin protein in the mouse embryonic fibroblasts REV1KO cells compared to the WT controls. The graph represents quantified data from **Fig. 4B**. Results are shown as mean ± S.E.M. (N = 3 biological replicates). **P*<0.01 values calculated by ***unpaired Student's t-test***. Statistical test done using Graphpad Prism 10.
